# Importance of Diagnostic Accuracy in Big Data: False-Positive Diagnoses of Type 2 Diabetes in Health Insurance Claims Data of 70 Million Germans

**DOI:** 10.3389/fepid.2022.887335

**Published:** 2022-05-23

**Authors:** Ralph Brinks, Thaddäus Tönnies, Annika Hoyer

**Affiliations:** ^1^Chair for Medical Biometry and Epidemiology, Faculty of Health, School of Medicine, Witten/Herdecke University, Witten, Germany; ^2^Institute for Biometry and Epidemiology, German Diabetes Center, Düsseldorf, Germany; ^3^Biostatistics and Medical Biometry, Medical School OWL, Bielefeld University, Bielefeld, Germany

**Keywords:** Epidemiology, chronic diseases - epidemiology, mortality, prevalence, incidence, illness-death model, aggregated data, non-communicable chronic disease (NCD)

## Abstract

Large data sets comprising diagnoses of chronic conditions are becoming increasingly available for research purposes. In Germany, it is planned that aggregated claims data – including medical diagnoses from the statutory health insurance – with roughly 70 million insurants will be published regularly. The validity of the diagnoses in such big datasets can hardly be assessed. In case the dataset comprises prevalence, incidence, and mortality, it is possible to estimate the proportion of false-positive diagnoses using mathematical relations from the illness-death model. We apply the method to age-specific aggregated claims data from 70 million Germans about type 2 diabetes in Germany stratified by sex and report the findings in terms of the age-specific ratio of false-positive diagnoses of type 2 diabetes (FPR) in the dataset. The FPR for men and women changes with age. In men, the FPR increases linearly from 1 to 3 per 1,000 in the age group of 30–50 years. For age between 50 and 80 years, FPR remains below 4 per 1,000. After 80 years of age, we have an increase to approximately 5 per 1,000. In women, we find a steep increase from age 30 to 60 years, the peak FPR is reached at approximately 12 per 1,000 between 60 and 70 years of age. After age 70 years, the FPR of women drops tremendously. In all age groups, the FPR is higher in women than in men. In terms of absolute numbers, we find that there are 217,000 people with a false-positive diagnosis in the dataset (95% confidence interval, CI: 204–229), the vast majority being women (172,000, 95% CI: 162–180). Our work indicates that possible false-positive (and negative) diagnoses should appropriately be dealt with in claims data, for example, by the inclusion of age- and sex-specific error terms in statistical models, to avoid potentially biased or wrong conclusions.

## Introduction

Aggregated data about the prevalence and incidence of chronic conditions become more and more available for research purposes. Usually, such data refer to a survey period and are presented aggregated in age- and sex strata. A prominent example is the National Health And Nutrition Examination Survey (NHANES) conducted by the National Center for Health Statistics at the Centers for Disease Control and Prevention (1). For public health research, NHANES surveys health and nutritional data from the U.S. general population since 1971. Another example is the Global Health Data Exchange catalog comprising three decades of data about the most prevalent and severe diseases from all over the world (2). Regional databases may contain health data from millions of people. In Germany, for instance, it is planned that aggregated claims data including medical diagnoses from the statutory health insurance with roughly 70 million insurants will be published regularly (3). Given a large number of study participants at possibly many points in time, the validity of the diagnoses in such big datasets can hardly be assessed. By validity of diagnoses, we refer to two types of errors that may occur: on the one hand, people with the chronic condition, in reality, might not have the diagnosis coded in the dataset and can be assumed to be false negatively coded. On the other hand, people without the chronic condition, in reality, might have a corresponding diagnosis in the dataset. Henceforth, we refer to these as false-positive findings in the dataset. By opposing the diagnoses coded in the dataset with “reality,” that is, the “gold standard,” such as a medical diagnosis based on an extensive examination of a specialist, the diagnosis codes in the dataset can be interpreted similarly as a diagnostic test Table 1 shows the possible combinations of disease status according to the gold standard and a coded diagnosis in the dataset.

**Table 1:** Possible combinations of disease status and coded diagnoses in the data set.

	**Gold standard**	
Claims data	Diseased	Not diseased
Diagnosed	True positive	False positive
Not diagnosed	False negative	True negative

Given aggregated data about age-specific prevalence, incidence, and mortality, we use a recently proposed method to estimate the false-positive ratio (FPR). The core idea is to relate the temporal change of the prevalence with the incidence and the mortality information by a partial differential equation (PDE) (4). To make the PDE consistent with the empirically observed prevalence, incidence, and mortality data, FPR and false-negative ratio (FNR) of the data are needed (5). With the assumption that the FPR and FNR in the prevalence and incidence data are the same, we can estimate the FPR in a claims dataset comprising type 2 diabetes status in 70 million Germans (85% of the overall population). This allows us to estimate the number of people with a false-positive diagnoses of type 2 diabetes in the claims data.

## Methods

Before we describe how to estimate the FPR in the claims data, we briefly introduce the methodological approach. Based on the illness-death model for chronic conditions (4), we could derive a PDE that relates the temporal change of the age-specific prevalence *p* = *p*(*t, a*), that is, the proportion of people aged *a* at calendar time *t* with the chronic condition, with the incidence rate *i*(*t, a*), general mortality *m*(*t, a*), and the mortality rate ratio *R* = *R*(*t, a*).


(1) $$\begin{array}{l} \left(\partial_{\text{t}}+\partial_{\text{a}}\right)\text{p}=\left(1\;-\text{ p}\right)i\;-\text{ m }\times \;\text{p }\left(\text{R }-\;1\right)/\left[1\;+\;\text{p }\left(\text{R }-\;1\right)\right] \end{array}$$

The mortality rate ratio *R* is the quotient of the mortality rates *m*_1_(*t, a*) and *m*_0_(*t, a*) of people with and without the chronic condition, respectively, that is, *R*(*t, a*) = *m*_1_(*t, a*)/*m*_0_(*t, a*). **Equation 1** holds true for the true prevalence *p* and incidence rate *i*. If we assume an observed prevalence *p*^(obs)^ and an observed incidence *i*^(obs)^ in the dataset (possibly imperfect with respect to diagnostic accuracy), the true prevalence and incidence can be obtained from Equations (2a), and (2b) using the sensitivity (*se*) and specificity (*sp*).


(2a) $$\begin{array}{l} \text{p}=\left({\text{p}}^{\left(\text{obs}\right)}-1+{\text{sp}}_{\text{p}}\right)/\left({\text{se}}_{\text{p}}+{\text{sp}}_{\text{p}}-1\right)\; \end{array}$$

and


(2b) $$\begin{array}{l} \text{i}=\left({\text{i}}^{\left(\text{obs}\right)}-1+{\text{sp}}_{\text{i}}\right)/\left({\text{se}}_{\text{i}}+{\text{sp}}_{\text{i}}-1\right). \end{array}$$

In Equations (2a), and (2b), sensitivity (*se*) and specificity (*sp*) of the age-specific prevalence and incidence (indicated by the sub-index) need not necessarily be the same. In datasets where prevalence and incidence stem from different sources, for example, different samples or surveys, the distinction might still be useful. In this study, we assume that the data generating process of prevalence and incidence are the same, such that we can skip this distinction and assume *se*_p_ = *se*_i_ and *sp*_p_ = *sp*_i_ for all age groups *a*.

Given the observed prevalence *p*^(obs)^, observed incidence *i*^(obs)^, general mortality *m*, mortality rate ratio *R*, and sensitivity *se* = *se*_p_ = *se*_i_, we can insert **Equations 2a** and **2b** into **Equation 1** to estimate the specificity *sp* = *sp*_p_ = *sp*_i_ (5). Thus, for known sensitivity *se*, we can calculate *sp* = 1 – *FPR* from these data by a functional relation Φ:


(3) $$\begin{array}{l} \text{sp}=\Phi \left(\text{se},{\text{p}}^{\left(\text{obs}\right)},{\text{i}}^{\left(\text{obs}\right)},\text{m},\text{R}\right). \end{array}$$

The exact formula for the functional relation Φ between *sp* on the left-hand side and *se*, *p*^(obs)^, *i*^(obs)^, *m*, and *R* on the right-hand side of **Equation 3**, is lengthy and presented together with its derivation in the supplement of Ref. (5).

Usually, we do not know the sensitivity *se* of the diagnoses in the dataset. To overcome this problem, we use a probabilistic approach as in (5) and randomly sample *se* from epidemiologically reasonable ranges between 50 and 99.9%. This does not impose a problem, because the functional relation as in Equation (3) is robust with respect to *se*, which has been demonstrated in (5). We examine how the estimated specificity *sp* changes and present the result as false positive ratio *FPR* = 1 – *sp*. The FPR is allowed to vary over age, independently for men and women in relevant age range 25 to 85 years. The algorithm requires that the age resolution, i.e., the difference between two consecutive age groups, is coarser than the temporal distance between the two prevalence surveys.

The algorithm described above is applied to the claims data about type 2 diabetes presented in Ref. (6). The claims data comprises approximately 70 million people during the period from 2009 to 2015. The number of people with a diagnosed type 2 diabetes are 5.8 and 6.1 million in 2009 and 2015, respectively. Prevalence of type 2 diabetes in men and women in these years is reported in 17 age groups (<15, 15-19, 20-24, …, 80-84, 85-89, 90+ years). Incidence rates for men and women are reported for the years 2012, 2013, and 2014 aggregated in five age groups (<20, 20-39, 40-59, 60-79, 80+ years). In the first step, reported prevalence *p*^(obs)^ and incidence *i*^(obs)^ are transformed by applying the *logit* function and the natural logarithm (*log*), respectively. Then, the transformed values are fit by the least squares method using a natural spline (*ns*) for age *a* with interaction terms in time *t* and sex *s*, that is, *y* ~ *ns*(*a*)^*^t^*^s where *y* refers to *logit*(*p*^(obs)^) and *log*(*i*^(obs)^), respectively. Note that we only have aggregated data, which prohibits more sophisticated statistical methods such as negative binomial regression. The degrees of the natural splines for the transformed reported prevalence and incidence are determined based on the number of available data points and visual comparison of the fitted functions with the reported input prevalence and incidence data.

For applying the functional Φ as in **Equation 3**, the general mortality *m* and the mortality rate ratio *R* are required. The general mortality is taken from the Human Mortality Database (8). The mortality rates of men and women in Germany during the 5 years period 2010-2014 are fit by a polynomial of degree two in age *a* to the logarithmized mortality rates in the age range 15–95 years. Impact of sex *s* was implemented by an interaction term, that is, the model equation reads *log*(*m*) ~ (*a*^2^ + *a*)^*^s. The degree of the polynomial was chosen by visual inspection of the fitted function with the input mortality rates. The age-specific mortality rate ratios *R* for men and women refer to the year 2014 and stem from the National Diabetes Surveillance report at the Robert Koch Institute (7). After application of a log-transformation, a natural spline in age *a* has been fit to *R*. Sex *s* is taken into account by an interaction term. Thus, the model reads *log*(*R*) ~ *ns*(*a*)^*^s. The degree of the natural spline is again determined based on the number of available data points and visual comparison of the fitted functions with the reported mortality rate ratios *R*.

After these data input and fitting routines, **Equation 3** is applied and the associated age-specific FPRs for men and women are calculated. Since the prevalence data are given in 2009 and 2015, the temporal difference is 2015–2009 = 6 years, and estimates for age groups more than 6 years apart are possible. We chose ages to be *a* = 25, 32.5, 40, …, 77.5, 85.

To estimate the absolute number of people with a false-positive diagnosis of type 2 diabetes, we interpolated the *FPR*, the corrected prevalence *p* [according to **Equation 2a**], and the number of people *N* in the claims data with their age-distribution to all age groups from 20 to 100 years. Then, the number of people *N*^(fp)^ with a false-positive diagnosis is calculated by 4.


(4) $$\begin{array}{l} {\text{N}}^{\left(\text{fp}\right)}=\overset{100}{\underset{\text{a}=20}{\sum }}\text{S}\left(\text{a}\right)\times \text{FPR}\left(\text{a}\right) \end{array}$$

where *S*(*a*) is the estimated number of people aged *a* without type 2 diabetes *S* = (1 – *p*) × *N*.

Since we sampled 100,000 sensitivity values, we obtained a large number of estimates for FPR in men and women using **Equation 3**. Accordingly, **Equation 4** provides a random distribution of possible values in men, women, and total. Empirical quantiles (2.5, 50, and 97.5%) for the resulting distributions are reported.

All calculations are performed in the free statistical software R, version 4.1.0 (The R Foundation for Statistical Computing). The source code and data for running the analysis have been published in the open-access repository Zenodo with digital object identifier (DOI) 10.5281/zenodo.5906275 (9). The data from the Human Mortality Database are available after registration only (8). We respect this policy and do not upload the raw mortality data to the Zenodo repository. Instead, in the uploaded source code we present the fitted coefficients of the regression model for the mortality rates. Using the coefficients instead of the raw data, which the coefficients were derived from, guarantees that the code is fully functional without unveiling data protected under a policy. Of course, using the coefficients from the regression model does not affect any of the conclusions drawn in this work, because the results are identical.

## Results

**Figure 1:**
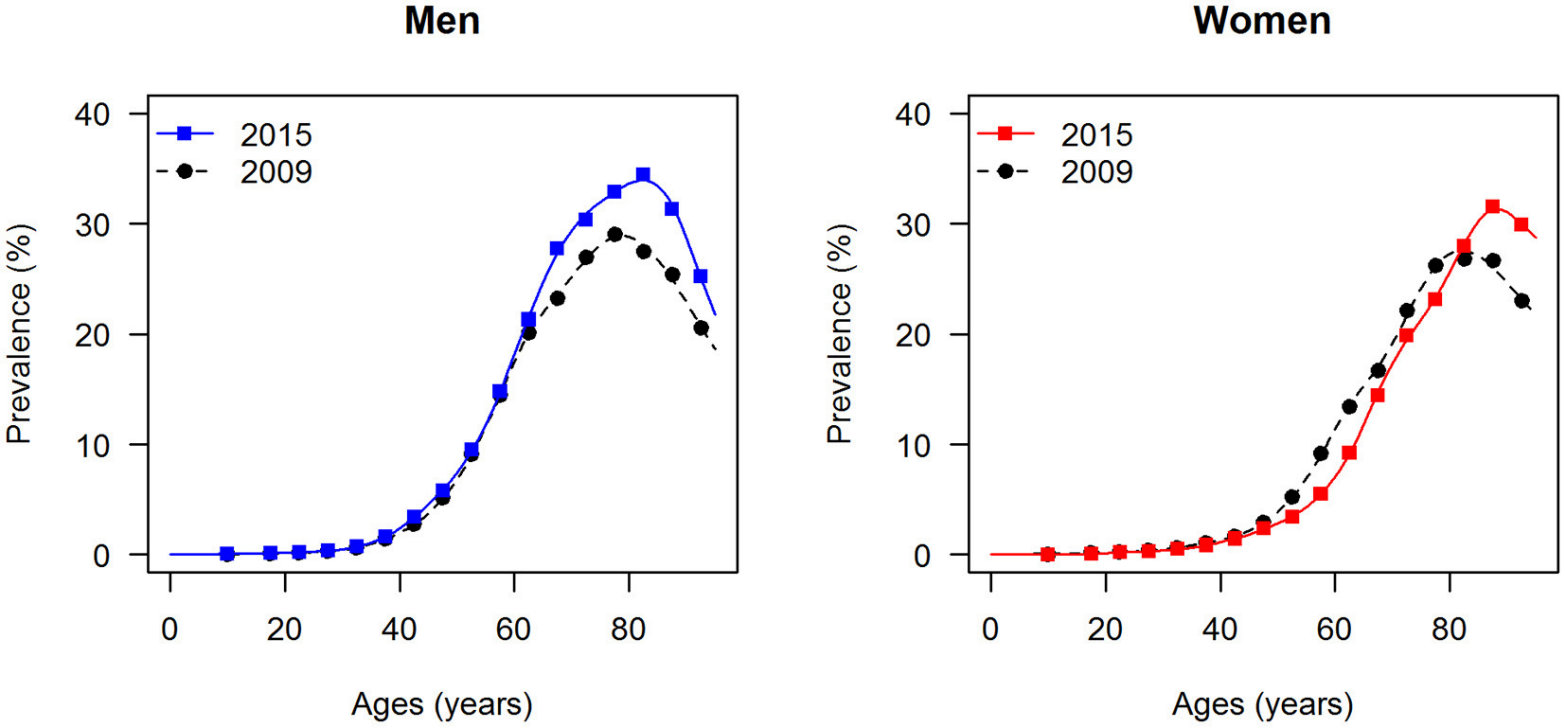
Age-specific prevalence of type 2 diabetes for men (left panel) and women (right panel) in the years 2009 and 2015. The data points and curves are the reported values from the claims data (6) and the fitted functions (lines), respectively.

**Figure 2:**
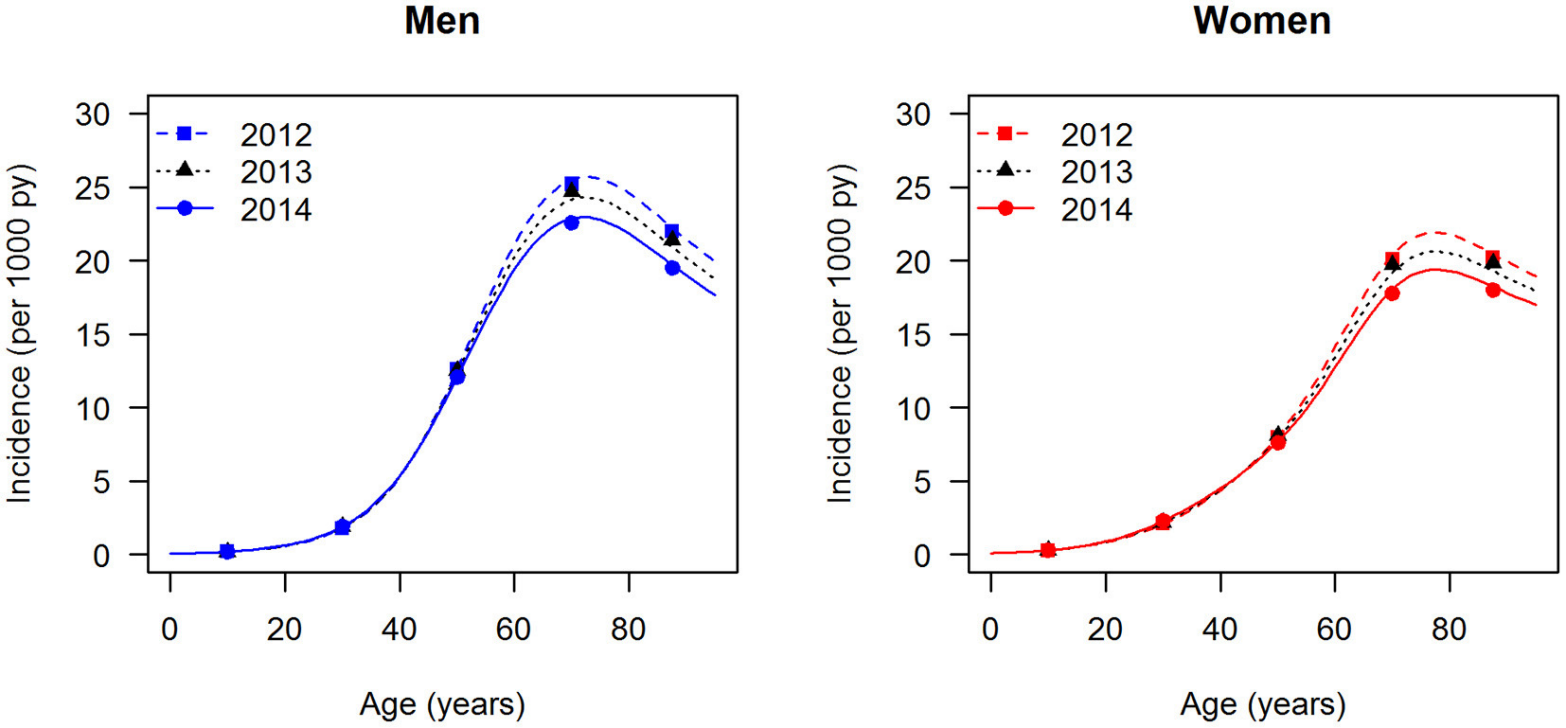
Age-specific incidence of type 2 diabetes for men (left panel) and women (right panel) in the years from 2012 to 2014. The data points and curves are the reported values from the claims data (6) and the fitted functions (lines), respectively.

**Figure 3:**
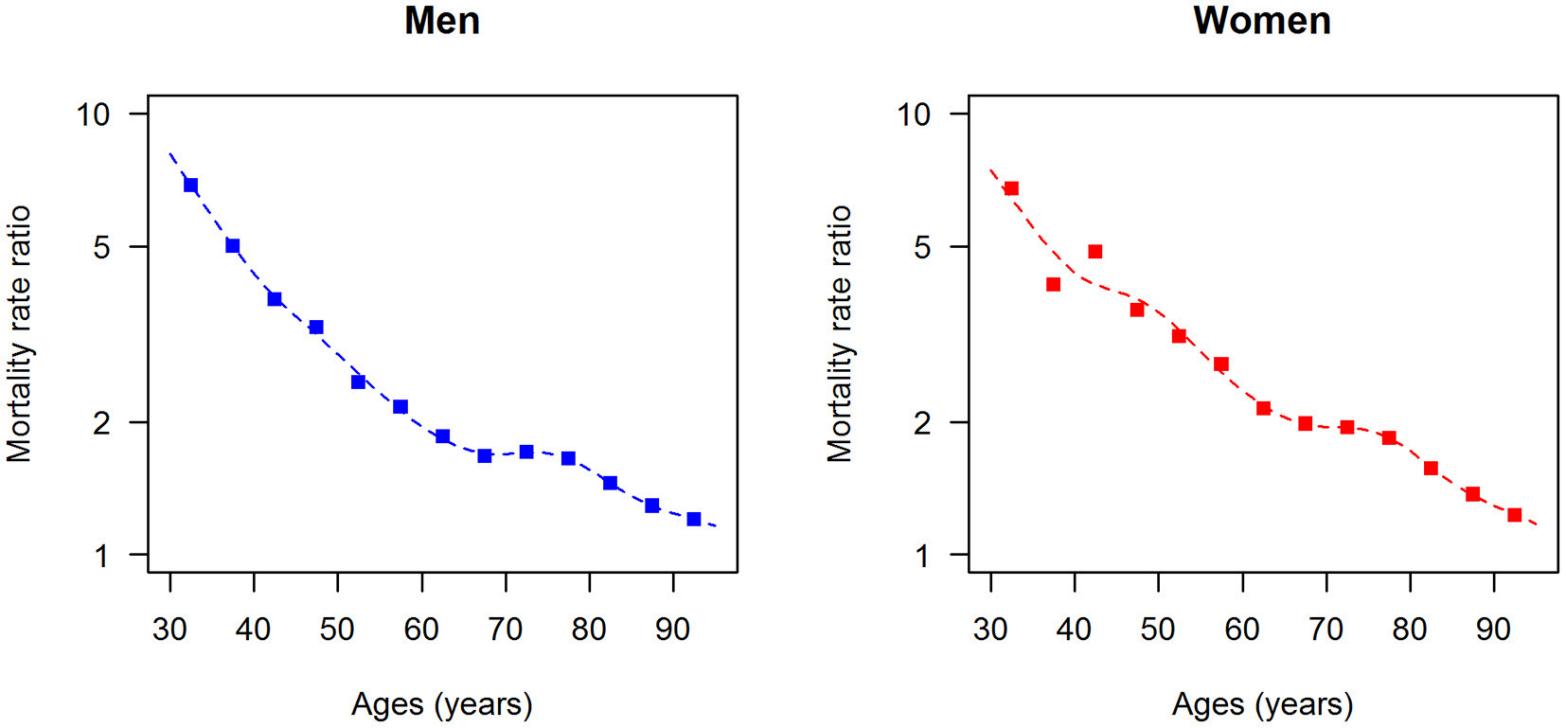
Age-specific mortality rate ratio (diabetes over non-diabetes) for men (left panel) and women (right panel) in the year 2014. The data points and curves are the reported values from the claims data (7) and the fitted functions (lines), respectively.

**Figure 4:**
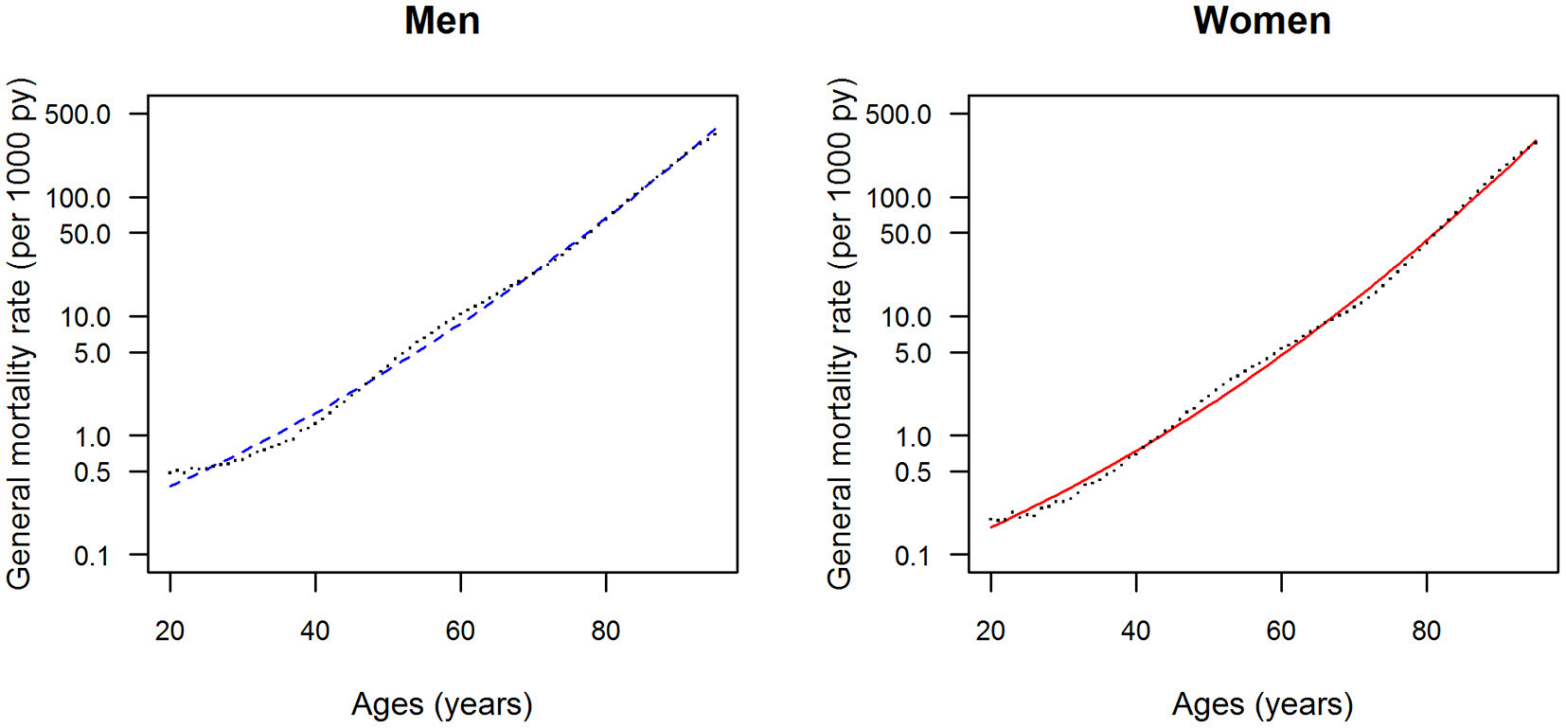
Age-specific general mortality rate for men (left panel) and women (right panel) in the years from 2010 to 2014. The data points and curves are the reported values from the Human Mortality Database (8) and the fitted functions (lines), respectively.

The data points in Figures 1, 2 show the reported prevalence *p* and incidence *i*, respectively, from the claims data, separately for men (left panel) and women (right panel) (6) These are opposed to the fitted curves (lines) after applying the logit and log transform to the data points. Similarly, in Figures 3, 4 the reported mortality rate ratios *R* and general mortality *m*, respectively, for men (left panel) and women (right panel) are shown together with their fitted curves (lines).

**Figure 5:**
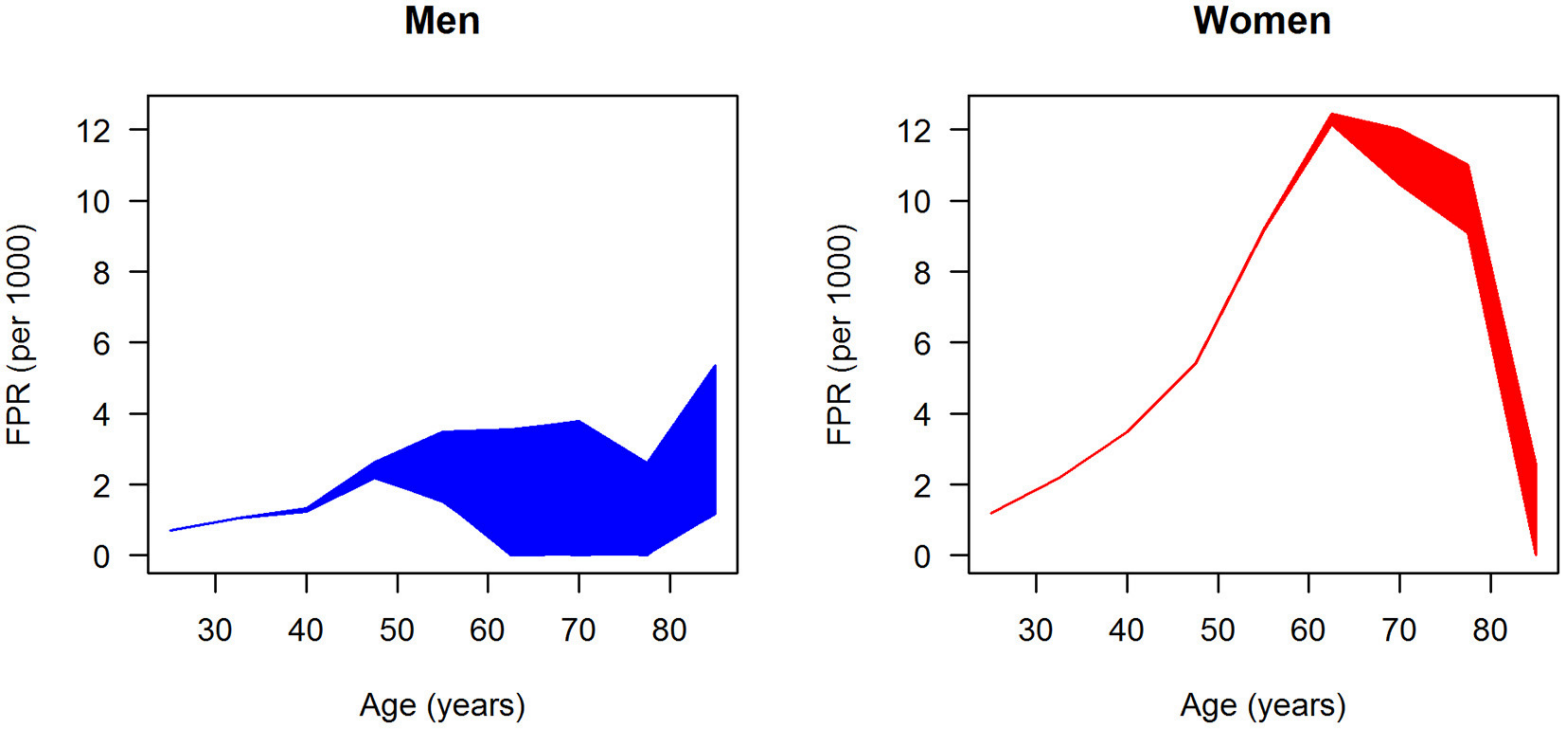
Age-specific ratio of false-positive diagnoses (FPR) in men (left panel) and women (right panel); 100,000 (random) scenarios about the age-specific sensitivity are simulated, and each line represents the FPR generated by one of these scenarios.

After fitting the input data, that is, prevalence, incidence, mortality rate ratios, and general mortality, we have all data at hand to estimate the age-specific FPR for men and women. For both sexes, 100,000 random samples of the sensitivity *se* are drawn uniformly from the range 50–99.9%, and the associated FPRs were calculated by **Equation 3**. The results are shown in Figure 5. Each of the 100,000 age-specific FPRs for men (left panel) and women (right panel) are depicted as a line, which at higher ages yield the impression of forming an area of possible values, blue and red, for men and women, respectively. In men, the FPR is <6 per 1,000 for all ages. In age groups below 50 years, the FPR in men increases linearly to approximately 2.5 per 1,000. At ages greater than 50 years, the maximum possible FPR is plateauing with a slight dip at age 77.5 years followed by an increase to approximately 6 per million. In women, the age-specific FPR steeply increases until age 60 years and peaks at about 12 per 1,000. For ages greater than 60 years, the FPR of women is decreasing again. In all age groups, the FPR is higher in women than in men.

**Figure 6:**
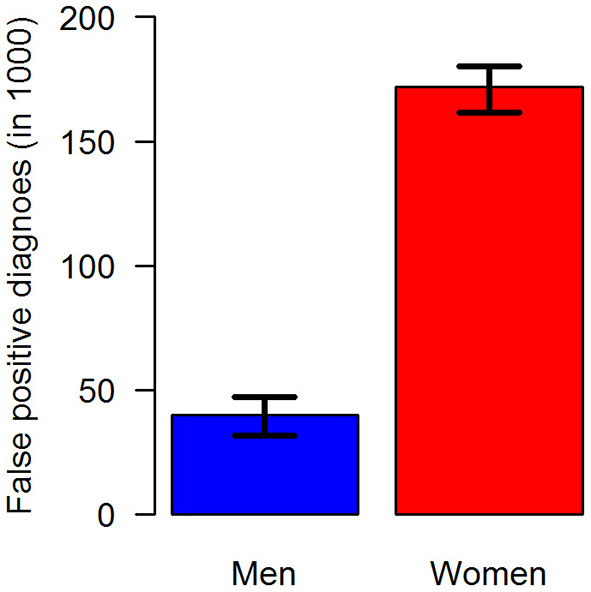
Number of false-positive diagnoses of type 2 diabetes in the claims data stratified by sex: men (blue, left) and women (red, right). The black antennas represent 95% confidence intervals.

**Figure 7:**
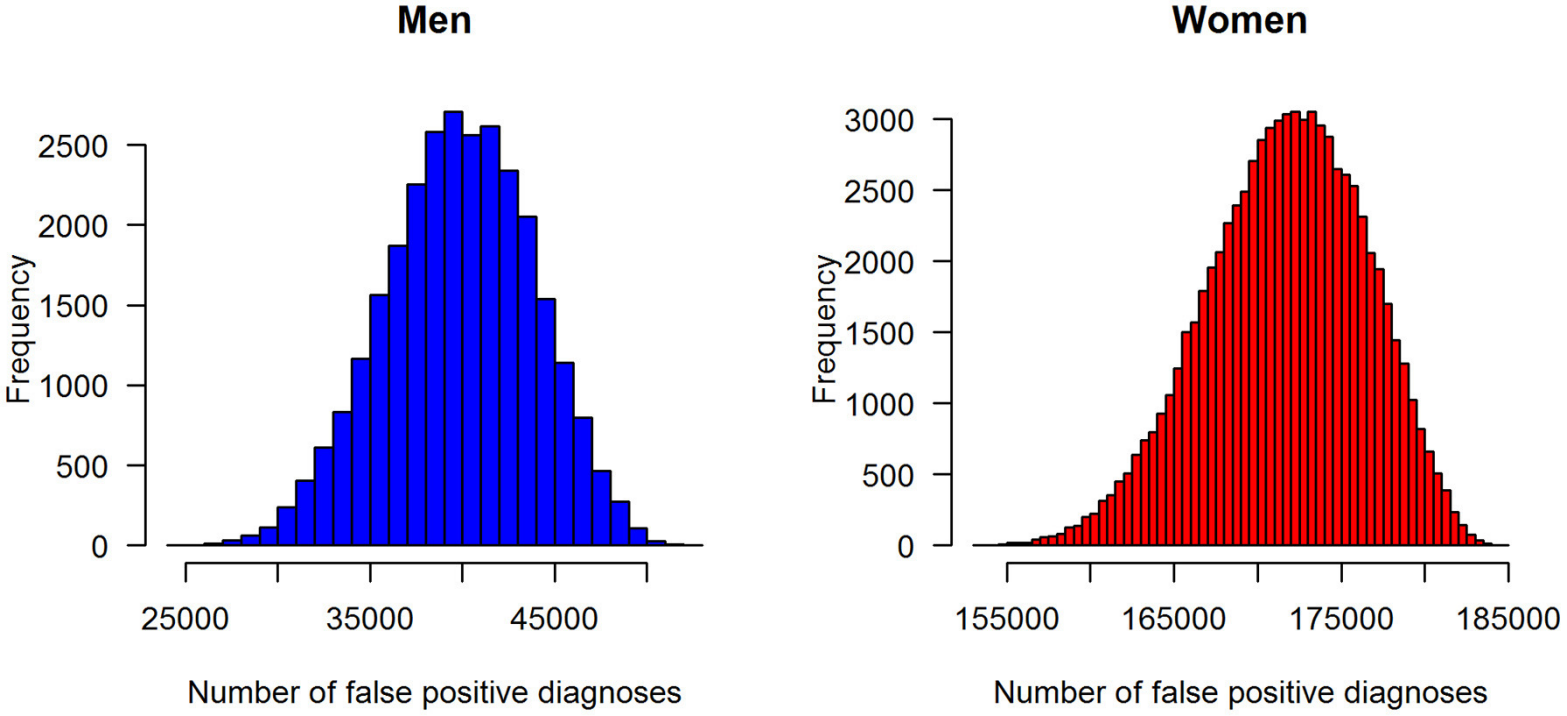
Distributions of the number of false-positive diagnoses of type 2 diabetes in the claims data stratified by sex: men (left panel) and women (right panel).

In terms of absolute numbers of false diagnoses in men and women, we obtained the median and 95% confidence bounds from the 100,000 random samples as reported in Table 2. For a better visual comparison of men and women, medians and 95% confidence intervals are presented in Figure 6. The associated empirical distributions of the 100,000 random samples are shown in Figure 7.

**Table 2:** Number of patients with falsely diagnosed type 2 diabetes in the claims data of approximately 70 million people in Germany.

	**Number of patients with false diagnoses of type 2 diabetes (in thousands)**	
	**Median**	**95% confidence interval**
Men	39.9	31.6 to 47.3
Women	172	162 to 180
Total	217	204 to 229

Overall, the vast majority of people wrongly diagnosed with type 2 diabetes in the claims data are women.

## Discussion

In this work, we estimated the age-specific FPR of type 2 diabetes in German women and men in a huge claims database. We used a mathematical relation between prevalence, incidence, and mortality for chronic conditions. To balance this relation, false-positive and false-negative diagnoses in the claims data need to be considered. Usually, the amount of false-positive and false-negative diagnoses is not accessible in such data. However, as the false-positive findings dominate the impact of false-negative findings by magnitudes, making coarse assumptions about the percentage of false-negative findings allows to examine FPRs.

We found that the age-specific FPR of men and women differed substantially. Across all age groups, FPR in men is lower than in women at the same age. At 60 years of age, the FPR of women is at least three times as high as the FPR of men. An anonymous reviewer pointed out that the FPR of women increases drastically due to around peri-menopausal age and thereafter. Hormonal changes during menopause increase the risk of several diseases (10) and clinicians are recommended to recognize early signs and symptoms of the menopause transition for accurate diagnoses and management (11). As a consequence of the difference between women and men, across all age groups approximately 172,000 women and 40,000 men have a false-positive diagnosis in the claims data. Reasons for the differences can only be speculated about. For example, in Germany, women are known to visit a physician more frequently than men (12). It seems plausible that less-frequent contacts decrease the probability of making a false-positive diagnosis in the claims data.

Reports about false-positive diagnoses of type 2 diabetes in huge databases are rare. More than a decade ago, a project about quality improvement for diagnoses of type 2 diabetes using computerized algorithms, found a percentage of false-positive diagnoses of approximately 5% in primary care patient records (13), which is similar to the percentage of 3.7% found in this study (217,000/5,800,000 = 0.037). The authors conclude their report with the note that the current practice of coding diabetic diagnostic data probably overestimates the prevalence of diabetes. We come to the same conclusion in the claims data examined in this study but should remind ourselves of the huge number of people with undiagnosed diabetes in Germany. In a representative population survey 2008-2011, the prevalence of diagnosed and undiagnosed diabetes in the overall population has been estimated to be 7.2, and 2.0%, respectively (14). Applying these findings to the claims data, we (roughly) estimated 70 million × (100–7.2%) × 2.0% = 1.3 million people with a missing diagnosis of type 2 diabetes in the claims data. Compared to the estimated 190,000 people with a false-positive diagnosis, false-negative diagnoses (undiagnosed) are a greater problem in the claims data than false-positive findings. Unfortunately, we have not found any report about the differences of false-positive diagnoses of type 2 diabetes between men and women.

This work mainly addresses the question of how (large) secondary data can be used for epidemiological analyses. Frequently, claims data are easily accessible for large populations. Thus, concluding relies on large numbers of cases, which seemingly provides enormous statistical power and a large potential for scientific analyses. However, it is clear that claims data and diagnoses within these data are collected for non-scientific purposes such as documentation and reimbursement. Although making faulty diagnoses, for example, coding a tentative diagnosis as an ascertained one, is considered fraud by national law, it is clear that scientific quality criteria are rarely met. In the claims data about type 2 diabetes, there is no difference between the diagnoses of general practitioners and specialists. Validation studies on individual patient level conclude that diagnoses in claims data have contextual problems, which requires careful and critical analysis (15). Our analysis provides insights into diagnostic accuracy, especially into the amount of false-positive diagnoses of type 2 diabetes.

Our method has several advantages. First, the approach described in this study can be applied to other chronic diseases and requires aggregated data only. Hence, the method may be used in a variety of settings where individual data are hard to obtain, for example, when strict data protection rules apply. Second, the method is flexible about the data sources. Data about the general mortality may frequently be obtained from the national statistical offices. If, furthermore, only prevalence and incidence data are available, the missing data about the mortality rate ratio might be taken from comparable populations, where it is available. An old argument states that the mortality rate ratio provides a stable measure in a wide variety of human populations (16).

Another advantage of the analysis presented in this study may be seen in the fact that the data used refer to the same population, that is, insurants of the statutory health insurance in Germany, and to a similar period (2009-2015). Using them in the same analysis seems reasonable.

The approach described and used has several limitations. Irrespective of prevalence data or incidence data are considered, sensitivity and specificity are assumed to be the same for both types of data (*se*_p_ = *se*_i_ and *sp*_p_ = *sp*_i_ for all age groups). A justification for this assumption in the diabetes data analyzed in this study can be seen in the same origin of the underlying diagnoses that have been used to estimate prevalence and incidence. However, the case definitions for prevalent and incident cases differ slightly. In short, a prevalent case is defined as someone having two ascertained diagnoses of type 2 diabetes in the study year 2009 or 2015 (6). An incident case has been defined as someone who has two diagnoses of type 2 within a year during 2012-2014 but is without a diagnosis in the three preceding years. It is not guaranteed that these definitions are consistent in all aspects. For example, it might happen that someone registered as an incident case in 2014 might not be counted as a prevalent case in 2015. Here, we make the implicit assumption that these cases are rare. Usually, patient records are arranged in a way that incident cases of type 2 diabetes are counted as prevalent cases afterward. Unfortunately, we do not have the individual patient data available such that this assumption could not be assessed. In theory, the assumption of the same sensitivity and specificity in both types of data can be released by applying **Equations 2a** and **2b**, with *se*_p_ ≠ *se*_i_ and *sp*_p_ ≠ *sp*_i_.

Another limitation comes from the fact that the data used to estimate the mortality rate ratio (7) is not the same as the data used for prevalence and incidence (6). Although they refer to the same population (people covered by the German statutory health insurance system), the mortality rate ratio (*R*) is estimated on inpatient and outpatient diagnoses while prevalence and incidence refer to outpatient diagnoses only. One might think that for type 2 diabetes the differences are small, but strictly speaking, this has not been validated. Moreover, estimation of the mortality rate ratio has been accomplished irrespective of the problem of false-positive and false-negative diagnoses in that dataset. Thus, we implicitly assume that the estimates of the age-specific mortality rate ratios are not affected by imperfect diagnostic accuracy. Until now, a systematic examination of the quality of mortality estimates from these claims data is missing. The last drawback should be mentioned: prevalence, incidence, and mortality rate ratio are estimated on the roughly 70 million people within the statutory health insurance. The general mortality, however, refers to the overall population of Germany (82 million people). Recent analyses indicate that in age groups below 90 years, there are no differences between the age-specific mortalities between these groups, see Figure 2 in Ref. (17).

To sum up, we assessed the age-specific percentage of false-positive diagnoses of type 2 diabetes in men and women by applying a partial differential equation to claims data covering approximately 85% of the German population. We found differences between age groups and between sexes. In younger age groups, false-positive diagnoses are less probable than in older age groups. Women are affected more by false-positive diagnoses than men. Our findings underpin the importance of considering false-positive and false-negative findings in secondary health data.

## Data Availability Statement

The datasets and source codes for this study can be found in the open access repository Zenodo under digital object identifier (DOI) 10.5281/zenodo.5906275, Web-Link https://doi.org/10.5281/zenodo.5906275.

## Ethics Statement

Ethical review and approval was not required for the study on human participants in accordance with the local legislation and institutional requirements. Written informed consent for participation was not required for this study in accordance with the national legislation and the institutional requirements.

## Author Contributions

RB had the initial idea for this work, developed the source code, and drafted the manuscript. TT and AH critically discussed the ideas and revised the manuscript. All authors gave substantial intellectual contributions. All authors contributed to the article and approved the submitted version.

## Conflict of Interest

The authors declare that the research was conducted in the absence of any commercial or financial relationships that could be construed as a potential conflict of interest.

## Publisher's Note

All claims expressed in this article are solely those of the authors and do not necessarily represent those of their affiliated organizations, or those of the publisher, the editors and the reviewers. Any product that may be evaluated in this article, or claim that may be made by its manufacturer, is not guaranteed or endorsed by the publisher.
